# Re-engineering pharmacology teaching and research: Can we adopt translational approach?

**DOI:** 10.4103/0253-7613.69941

**Published:** 2010-10

**Authors:** S. K. Kulkarni

**Affiliations:** Emeritus Professor of Pharmacology, Panjab University, Chandigarh, Presently, Director, Bombay College of Pharmacy, Mumbai, India. E-mail: skpu@yahoo.com

In the early 1980s, many of the medical schools and health sciences universities in the West integrated the preclinical departments such as anatomy, physiology, and biophysics into a new and emerging discipline of cell biology to harness the rapid developments in biochemistry, molecular biology, and genetics. They also moved from conventional to organ-or disease-based teaching. This provided great dividends in terms of knowledge base of the next generation of physicians and training of biomedical scientists. It also helped them to develop new facilities for imparting training. Unfortunately, this has not happened in our institutions. We continue to follow the rigid and conventional path. As a result, core capabilities in cell biology, molecular or genome science, and their integration and subsequent development of knowledge base have not taken place.

The pharmacological science is at crossroads. The drug discovery and development require hardcore training in experimental pharmacology at molecular level, and the clinical development of drugs calls for greater clinical acumen to weigh risk *versus* benefit in assessing the new therapy. Pharmacologists use drugs as tools to study basic mechanisms in biological systems, be it an isolated tissue (bioassay), cell preparation (single cell preparation as in patch-clamp study) or *in vivo* study (employing a mouse or a rat), and on the other hand a physician or clinical pharmacologist would be keen in rational or evidence-based use of medicines or to know compliance or noncompliance of a therapy in relation to its clinical outcome. In either case, basic training in pharmacological sciences has to be strong.

It is increasingly felt that a radical change in teaching of pharmacology (about medicines) at undergraduate level is necessary to ensure that future generation of prescribers are well versed with the fundamental principles that underlie good clinical practice. It is unfortunate that experimental pharmacology (the so-called classical pharmacology) has disappeared from the undergraduate teaching in medical colleges, even though it still exists to certain extent in pharmaceutical curriculum. The postgraduate curriculum in pharmacology has also not changed in these years, even if it has, it is only cosmetic. Many of the postgraduate and doctorate degree holders in pharmacology seek employment in pharmaceutical industry, and there are many job opportunities in discrete areas of pharmaceutical operations, such as clinical research, pharmacovigilance, pharmacokinetics, systems biology, regulatory affairs, genome medicine, and experimental medicine besides the “omics.” Many of these disciplines are not taught at postgraduate level. There are many private academies that now offer some of these courses as weekend programmes, and many graduates in medicine, ayurveda, and pharmacy take them to get suitable jobs in industry. The postgraduate curriculum should incorporate these modules in their teaching programme. It is always a great value if these courses become a part of the postgraduate curriculum of a university degree.

Many years ago while delivering the Col. R. N. Chopra oration at the annual conference of Indian Pharmacological Society (IPS), I had highlighted about the challenges facing the drug discovery science and cautioned about the increasing gap in pharmacology training between India and the rest of the world. The situation is no better today. Out of the 197 papers (including reviews, full papers, short communications, and letters to editor) published in *IJP* during the past three years (2007–2009), only 28% were from medical colleges, 34% from pharmaceutical institutions, and rest from allied disciplines including some from abroad. If *IJP* is the face of IPS, the publications (the numbers and the quality) in its official organ reflect the status of pharmacology research in the country. This is seen particularly at a time when the Indian pharmaceutical industry is growing at the phenomenal rate, 17% growth in the last quarter of 2009 and the turnover expected to cross USD 20 billion in the coming years. India has been considered a strategic destination for global R&D (preclinical, formulation, and clinical developments). As per the McKinsey and Company projections, 2009, the emerging markets of BRIC nations (Brazil, Russia, India, and China) will account for nearly half of the growth in the global pharmaceutical industry. It is also expressed that one of the biggest challenges of comprehending and capturing these growth numbers (“Capturing India Advantage”) is the shortage of specialised talent or human resources, particularly in the areas of systems biology, clinical research, and healthcare delivery. Pharmaceutical graduates and trained pharmacologists have a great role to play in realising the “India Advantage.”

At the global level, research is moving forward with great pace and inevitably new areas of challenges will emerge. In a competitive world and globalised development, we cannot be left behind. We need to catch up and catch up fast. Unlike digital technology, biological sciences need greater inputs and greater understanding and grinding in basic sciences. If teaching and training across the wide range of areas in pharmacology are not initiated in the very near future, the next generation of Indian pharmacologists (and in turn, the prescribers) will have hard time to converse with their peers at a global level. We often complain that Indian research is not cited in the western publications, and it will continue to remain an enigma.

Someone said, “It is all gloom and doom” unless we move forward with vigour and imagination. The pharmacology educationists have to come together and define the path, line of action, and timelines to achieve it. Research needs greater funding. It may not be possible for all the 300 plus medical colleges and even more number of pharmaceutical institutions to have similar kind of facilities for research. We need to identify individuals and institutions of potential to take up the challenge and support them with funding and freedom to develop with clear mandate to achieve the goals. Any half-hearted approach will leave the developmental process in a greater lurch.

## Translational approach: Can we adopt?

Recently, the National Institute of Health (NIH), Bethesda, USA has prepared a road map for medical research to re-engineer the existing departments of therapeutics into a new discipline of clinical and translational science. NIH has created a consortium which initially began with 12 health universities and expanded to 46 in 2009. It hopes to link 60 institutions by 2012 to energize the discipline of clinical and translational science. The broad objectives of the consortium include: (1) to captivate, advance, and nurture a cadre of well-trained multi- and interdisciplinary investigators and research team, (2) to create an incubator for innovative research tools and information technologies, and (3) to synergize multi- and interdisciplinary clinical and translational research and researchers to catalyze the application of new knowledge and techniques to clinical practice at the front lines of patient care. At the core of translational research, scientific discoveries are to be translated into practical applications to improve human health. In other words, translational research, “bench to bedside” or “mouse to man” is a two-way path aimed at reducing the barriers between basic and clinical research. The translation of the information generated in molecular and genome research to clinical science and back again to bench is a challenging task. The translational science can work as a powerful process, if harnessed, to drive the clinical research engine. All stakeholders, institutions, investigators, clinicians, professional bodies, and industries are expected to actively participate and support the growth of this innovative discipline.

The Ministries of Health and Family Welfare and Human Resource Development of Government of India are in the process of setting up of two councils, namely National Council for Human Resource in Health (NCHRH) and National Council for Higher Education and Research (NCHER) to administer health education and higher education in general. It should become the mandate of NCHRH (Health) to revamp the teaching and learning across all the disciplines of healthcare to address the pitfalls in the existing curriculum so that there is a free and conducive environment for learning and doing research in a multi- and interdisciplinary approach. Translation science may prove to be a right remedy not only for multi- and inter-disciplinary learning, but also for integrating traditional medicine with modern medical science. If we do not re-engineer the basic medical sciences now, departments including pharmacology will lose the relevance of the discipline.

